# Bat–parasite interaction networks in urban green areas in northeastern Brazil

**DOI:** 10.1017/S0031182022001718

**Published:** 2023-03

**Authors:** Rayanna Hellem Santos Bezerra, Adriana Bocchiglieri

**Affiliations:** Universidade Federal de Sergipe, Programa de Pós-graduação em Ecologia e Conservação, Laboratório de Mastozoologia, Cidade Universitária Prof. José Aloísio de Campos, São Cristóvão, SE, Brazil

**Keywords:** Centrality, Chiroptera, Diptera, modularity, urbanization

## Abstract

Interaction networks can provide detailed information regarding ecological systems, helping us understand how communities are organized and species are connected. The goals of this study were to identify the pattern of interaction between bats and ectoparasites in urban green areas of Grande Aracaju, Sergipe, and calculate connectance, specialization, nesting, modularity and centrality metrics. Bats were captured using 10 mist nets inside and on the edges of the fragments, and the collected ectoparasites were stored in 70% alcohol. All analyses were performed using R software. The interaction network consisted of 10 species of bats and 13 ectoparasites. Connectivity was considered low (0.12). The specialization indices for ectoparasites ranged from 0.50 to 1.00, and the value obtained for the network was 0.96, which is high. The observed nesting metric was low (wNODF = 1.47), whereas the modularity was high (wQ = 0.74), indicating that the studied network had a modular topology. All centrality metrics had low values. The observed modularity may have been caused by the evolutionary history of the bats and ectoparasites involved and the high specificity index of the interactions. The low centrality values may be associated with low connectivity and a high degree of specialization. This study provides relevant information on bat–parasite interactions in an urban environment, highlighting the need for further studies to improve our understanding of host–parasite interaction networks.

## Graphical abstract

**Figure:**
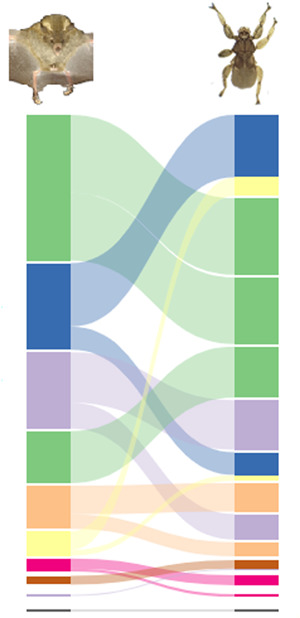

## Introduction

The order Chiroptera, represented by bats (Whitaker *et al*., 2009), has high diversity and wide geographic distribution; bats are found in different types of natural environments and urban areas (Reis *et al*., 2007). Similar to other animals, bats are subject to parasitism, and it is possible to observe numerous populations of mites, dipterans and ticks that feed on their hair follicles, body fluids or blood (Anderson and Ortêncio-Filho, 2006; Peracchi *et al*., 2011). When an ecological system is formed by several species interacting with each other, as in parasitism, it can be represented by networks. It is essential to consider all species as integral parts of ecological networks and not as isolated groups (Montoya and Raffaelli, 2010). Studying networks of interactions aids in the search for patterns of abundance, distribution and specialization of species, their degree of redundancy and their vulnerability to extinction (Tylianakis *et al*., 2007; Clayton *et al*., 2015; Zarazúa-Carbajal *et al*., 2016). In addition, networks can provide detailed information on ecological systems, helping elucidate how biological communities are organized, and how species are connected, underscoring the implications of these interactions for the ecosystem (Poulin, 2010; Freitas Júnior *et al*., 2020).

The network structure may differ according to the type of interaction represented (Bascompte, 2010; Thébault and Fontaine, 2010). For example, mutualistic interactions can be more nested, connected, characterized by a group of generalist species and less modular, whereas in antagonistic interactions, such as parasitism, there would likely be the formation of modules, and the species can be less connected (Thébault and Fontaine, 2010; Pires and Guimarães, 2013). Considering parasitism, the expected high modularity would result from the evolutionary history of the species and the high specificity of the interactions (Pires and Guimarães, 2013).

High specificity is a frequent feature in the relationship between bats and ectoparasites (Fritz, 1983; Giorgi *et al*., 2004), which can vary from 1 to several species and result from the adaptation to the host and phylogeny of the species involved (Balashov, 1984); the presence of the same parasite species on more than 1 host species may occur because of the inheritance of genetic characteristics (Poulin and Rohde, 1997). However, a nested pattern in the interaction between bats and their parasites has been recorded (Presley, 2007) and may be associated with the differences in species abundance or parasite adaptability (Lewinsohn *et al*., 2006; Canard *et al*., 2012).

Among the factors influencing the interaction network, changes in the landscape principally caused by habitat fragmentation and alteration are important aspects to be evaluated (Haila, 2002). For example, changes in habitat availability resulting from urbanization can directly affect the survival and structure of biological communities, altering the strength of interactions between species and the topology of networks (Patterson *et al*., 2009; Pilosof *et al*., 2012; Ferreira *et al*., 2013). Thus, aspects related to community structuring (richness, abundance and composition) resulting from environmental changes could result in changes in the interaction networks (Kruess, 2003; Tylianakis *et al*., 2007; Bartomeus *et al*., 2016).

Thus, the goals of this study were to represent, through a bipartite network, the interactions between bats and ectoparasites in the urban green areas of Grande Aracaju, Sergipe; identify the interaction pattern through network topology and calculate the metrics of connectance, specialization, nesting, modularity and centrality. We expected that the host–parasite interaction network presents a modular topology because of the high specificity of the parasites, with low connectivity and nesting values and high specialization and modularity values. We hypothesized that the most central species will be the most abundant in the study because of the greater probability of interactions.

## Materials and methods

### Study area

**Fig. 1.**
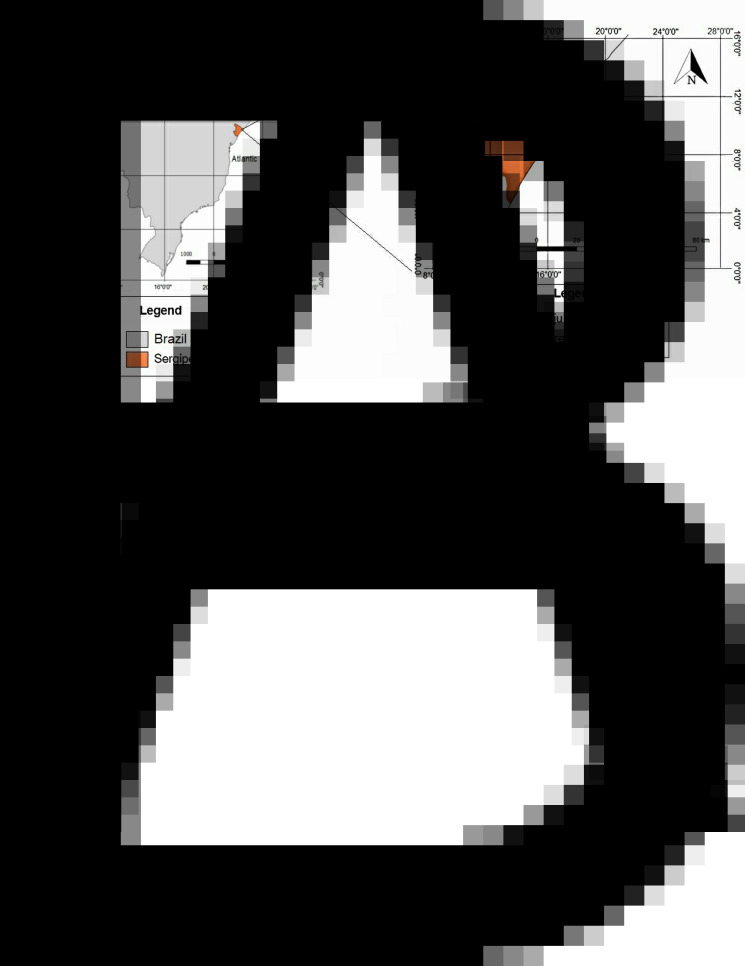
(a) Brazil with state of Sergipe in highlight; (b) the municipalities with indication of the urban green areas (UFS, SEFAZ and Vila) used for capturing bats located in Grande Aracaju, Sergipe.

The study was conducted in 3 urban green areas located in Grande Aracaju, Sergipe, northeastern Brazil (Fig. 1). The sampled areas belong to the Campus São Cristóvão da Universidade Federal de Sergipe (UFS; 10°55ʹ34.3ʺS; 37°06ʹ09.2ʺW), Secretaria de Estado da Fazenda de Sergipe (SEFAZ; 10°54ʹ38.8ʺS; 37°05ʹ27.9ʺW) and Vila Militar dos Oficiais do Exército (Vila; 10°55ʹ31.6ʺS; 37°03ʹ36.7ʺW). During the study period, the accumulated precipitation was 1773.4 mm, with an average temperature ranging from 24.7 to 29.3°C. The month with the lowest rainfall was December 2019 (8 mm) and the month with the highest rainfall was April 2020 (284.6 mm) (INMET, 2021).

The UFS area corresponded to a remnant of the Atlantic Forest, approximately 0.99 ha, and near a fragment of native forest to the Poxim river (Bezerra and Bocchiglieri, personal observation). This area is characterized by dense vegetation, closed canopies and intense artificial light. Despite being considered a remnant of the Atlantic Forest, the use of exotic species for afforestation of the UFS campus caused de-characterization of the natural environment (White *et al*., 2011), with approximately 34% of its vegetation being exotic (Gomes *et al*., 2017).

The SEFAZ is 1.58 ha with a plantation of bananas (*Musa* sp.), resulting in an area with low vegetation and a dense understory. Besides this plantation, the vegetation presents itself differently, having other plant species that occur more widely, with a less dense understory and closed canopy. This area is next to a soccer field and has intense public lighting on the surrounding streets. In addition, solid residue and dense litter were observed (personal observation).

In Vila, an area of 1.56 ha is characterized by the predominance of *Terminalia catappa* L. (almond trees), with a closed canopy. Despite corresponding to a residential area, the artificial lighting of houses close to the studied green area was less intense than that of the UFS. At the site, it is possible to observe large amounts of solid waste, construction rubble and dense litter (personal observation).

### Data collection and analysis

In the areas of UFS and SEFAZ, the campaigns were conducted in September and November of 2019, January, June, July, August, September and November of 2020 and January of 2021. In the Vila area, the campaigns were carried out in September and November of 2019, January and December of 2020 and January and February of 2021. These campaigns were carried out during 2 consecutive monthly nights in each area. Because of the COVID-19 pandemic, 9 campaigns were conducted at UFS and SEFAZ and 6 at Vila during this period.

For the capture of bats, 10 mist nets (9 × 3 m^2^, mesh 20 mm) were used in each area, arranged at the ground level. They were set from sunset to midnight and inspected at 30 min intervals. The bats were identified based on the method described by Reis *et al*. (2017) and posteriorly released at the same capture site. After capture, each bat was inspected with the naked eye for ectoparasites that, when found, were collected manually, stored in ‘Eppendorf’ microtubes with 70% alcohol and the data of each host were properly identified. The identification of the ectoparasite species was realized under a Bell stereomicroscope (SZT model) according to the keys proposed by Guerrero (1994*a*, 1994*b*, 1995) and Whitaker *et al*. (2009).

The data corresponding to the 3 sampled urban green areas were grouped for a more robust analysis. A weighted data matrix was used to generate the interaction network, where bats were represented in the rows, ectoparasites were represented in the columns and the interactions corresponded to the number of times an association was observed in different hosts, considering only the primary associations. The bipartite interaction network was generated using the ‘bipartiteD3’ package in R software (R Core Team, 2021).

Connectivity was determined based on the proportion of interactions occurring relative to the number of possible interactions (Mello *et al*., 2015). The specialization index, which indicates complementary specialization, was separately calculated for the ectoparasite species (*d*′) and the network as a whole (*H*_2_′), representing how sets of interactions between species differed from each other and varied from 0 (complete generalization) to 1 (complete specialization) (Blüthgen *et al*., 2006; Mello *et al*., 2015).

For nesting, the metric ‘nestedness metric based on overlap and decreasing fill’ (wNODF) was used for weighted matrices, where the values ranged from 0 (no nesting) to 100 (complete nesting) (Almeida-Neto and Ulrich, 2011). The QuanBiMo (wQ) algorithm was used for modularity, where values ranged from 0 (no modularity) to 1 (complete modularity) (Dormann and Strauss, 2014). To verify the significance of connectance, specialization, nesting and modularity, 1000 random networks were generated using the *vaznull* null model, which reorganizes the interactions while preserving the same marginal totals and connectance as the observed network (Vázquez *et al*., 2007).

Three widely used metrics were considered (relative degree, closeness centrality and betweenness centrality). The relative degree (Kr) corresponds to the number of interactions a species makes in relation to the total number of interactions it could make (Mello *et al*., 2015). The closeness centrality (CC) measures the proximity of a species to other species in the network, corresponding to the smallest number of links separating 2 species (Mello *et al*., 2015). Betweenness centrality (BC) measures the importance of a species as a connector between different parts of the network (Mello *et al*., 2015). Centralities were calculated only for the bat species. The graph generated for the centrality metrics was generated using Gephi software (Bastian *et al*., 2009). All analyses were conducted using R software with a significance level of 5% (R Core Team, 2021).

## Results

**Fig. 2.**
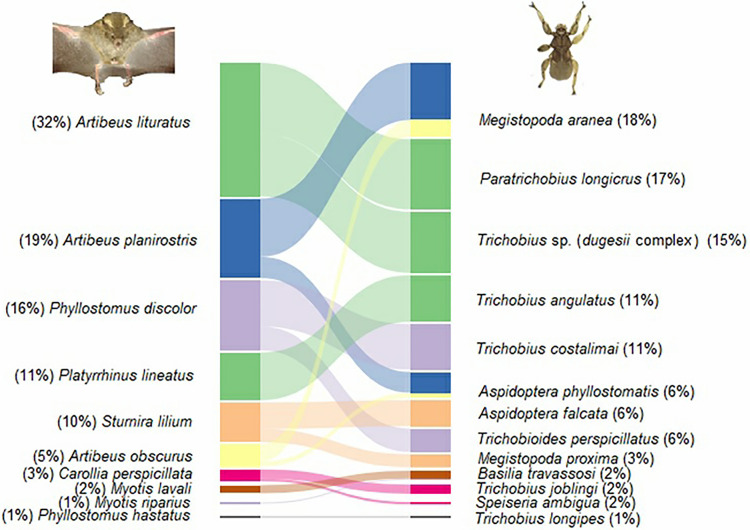
Interaction network between bats and ectoparasites captured in urban green areas of Grande Aracaju, Sergipe, Brazil. The lines and their different colours represent interactions between species, and the width of the line indicates the strength of the interactions. The values in parentheses indicate the frequency of occurrence of the species.

We captured 278 parasitized bats and 875 ectoparasites. The interaction network consisted of 10 species of bats belonging to the families Phyllostomidae (*S* = 8) and Vespertilionidae (*S* = 2) and 13 species of ectoparasites belonging to the families Streblidae (*S* = 12) and Nycteribiidae (*S* = 1; Appendix 1), totalling 16 interactions. The most representative bat species were *Artibeus lituratus* (Olfers, 1818) and *Artibeus planirostris* (Spix, 1823). The most abundant ectoparasites were *Megistopoda aranea* (Coquillett, 1899) and *Paratrichobius longicrus* (Miranda-Ribeiro, 1907) (Fig. 2).

The observed connectance for the bat–ectoparasite interaction network was 0.12, which was lower than expected by the null model (*C* = 0.53; *P* < 0.05). This result indicated that the species that made up the network had few interactions, and a maximum of 2 interactions per species was observed (Fig. 1). The values obtained for ectoparasites (*d*′) for the specialization index ranged from 0.50 to 1.00 (Table 1). For the entire network, the specialization value was 0.96, which was higher than expected (*H*_2_′ = 0.06; *Z*-score = 81.60; *P* < 0.05). Thus, the network was highly specialized, with most parasite species (77%) infesting only 1 host.

**Table 1.** Values referring to the complementary specialization index (*d*′) of the ectoparasites parasitizing bats captured in urban green areas of Grande Aracaju, Sergipe

Ectoparasites	*d*′
*Aspidoptera falcata*	0.85
*Aspidoptera phyllostomatis*	0.50
*Basilia travassosi*	1.00
*Megistopoda aranea*	0.82
*Megistopoda proxima*	0.67
*Paratrichobius longicrus*	0.64
*Speiseria ambigua*	0.65
*Trichobioides perspicillatus*	0.61
*Trichobius angulatus*	1.00
*Trichobius costalimai*	0.81
*Trichobius joblingi*	0.93
*Trichobius longipes*	1.00
*Trichobius* sp. (*dugesii* complex)	0.59

Nesting obtained for the network was extremely low (wNODF = 1.47), and differed from that expected by the null model (wNODF = 81.43; *Z*-score = −17.66; *P* < 0.05). For modularity, the value obtained was higher (wQ = 0.74) than that expected by the null model (wQ = 0.12; *P* < 0.05), indicating that the studied network had a modular topology, with the formation of 8 modules. Of the modules formed, only 2 were composed of more than 1 species of bats, *Artibeus obscurus* (Schinz, 1821) and *A. planirostris*, and *Myotis lavali* Moratelli, Peracchi, Dias & de Oliveira, 2011 and *Myotis riparius* Handley, 1960.

**Fig. 3.**
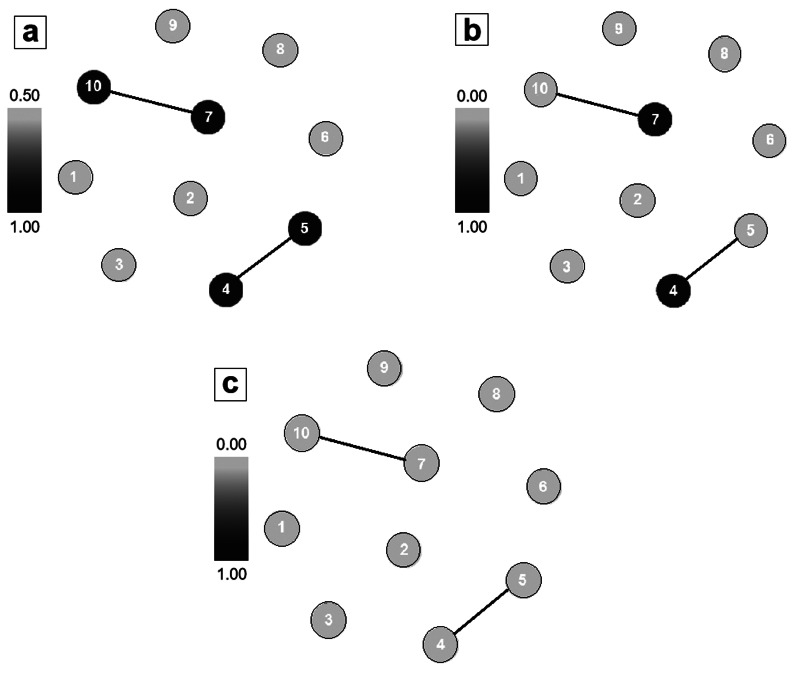
Unipartite projections of the original bipartite nets: the circles represent the bat species captured in urban green areas of Grande Aracaju, Sergipe. Brazil; 2 species are connected when they are parasitized by at least 1 common species of parasite. The stronger the colour, the greater the value of centrality observed [(a) relative degree, (b) closeness centrality and (c) betweenness centrality]. The numbers correspond to bat species (1: *Artibeus lituratus*, 2: *Phyllostomus discolor*, 3: *Sturnira lilium*, 4: *Artibeus obscurus*, 5: *Artibeus planirostris*, 6: *Carollia perspicillata*, 7: *Myotis lavali*, 8: *Phyllostomus hastatus*, 9: *Platyrrhinus lineatus* and 10: *Myotis riparius*).

Regarding centrality metrics, the relative degree ranged from 0.5 to 1.0, with the highest values for *A. obscurus*, *A. planirostris*, *M. lavali* and *M. riparius*. For CC, the values obtained ranged from 0.0 to 1.0, with the highest values corresponding to *A. obscurus* and *M. lavali*. In relation to BC, all values were equal to 0, indicating that in the studied network, no species was considered important for maintaining structure connections (Fig. 3).

## Discussion

The Phyllostomidae family corresponded to 51.38% of the species recorded in Brazil (Garbino *et al*., 2020) and was the most abundant family in this study. According to Pech-Canche *et al*. (2011), the capture method used in this study is highly effective in capturing representatives of this family. Richness of other families, such as Vespertilionidae, Molossidae and Emballonuridae, may have been underestimated as their representatives can fly higher and detect the mist net more easily (Simmons and Voss, 1998; Rinehart and Kunz, 2001). For ectoparasites, the high occurrence of *M. aranea* and *P. longicrus* could be associated with the high capture rate of their primary hosts, *A. planirostris* and *A. lituratus*, respectively (Graciolli and Rui, 2001). Species of the genus *Artibeus* are commonly recorded in urban areas, being considered opportunistic, with varied feeding habits and can benefit from fruit plants used in local afforestation, allowing their high occurrence in this type of environment (Zortéa and Chiarello, 1994; Barros *et al*., 2006; Lima, 2008).

The interaction network obtained in this study exhibited low connectance, low nesting, high general and complementary specialization and high modularity. Thus, the network topology was modular. Such a pattern has been reported for other host–parasite interactions (e.g. Poulin, 2010; Krasnov *et al*., 2012; Morris *et al*., 2014) and bats and their ectoparasites (Urbieta *et al*., 2021). This modularity may be caused by the evolutionary history of the bats and ectoparasites contributing to the high specificity index of the interactions (Pires and Guimarães, 2013).

The high degree of specialization and modular pattern observed in this study could be associated with the high specificity of ectoparasites, as 77% were parasitized by only 1 host species (monoxenes). For parasitism, the physical approach of the hosts is necessary, and it is believed that most of the parasites are monoxenes (Marshall, 1982; Dick *et al*., 2009) or stenoxenes (Dick, 2007), with this high specificity being a frequent characteristic of the relationship between parasites and bats (Fritz, 1983), as reported in other studies conducted in Sergipe (Bezerra *et al*., 2016; Bezerra and Bocchiglieri, 2018).

A modular network is characterized by species with few links that relate preferentially within a cohesive subgroup of species rather than other species in the network (Olesen *et al*., 2007; Krasnov *et al*., 2012). In the present study, only 2 groups formed by more than 1 bat species were observed, and these groups were formed by bats belonging to the same genus (*Artibeus* spp. and *Myotis* spp.). In modular networks, owing to high species specificity, modules are often created by phylogenetically related bat species with similar parasitic fauna (Mello *et al*., 2011; Krasnov *et al*., 2012; Freitas Júnior *et al*., 2020). According to Wenzel *et al*. (1966) and Marshall (1982), parasites belonging to the Streblidae and Nycteribiidae families are highly specific, and each fly species generally parasitizes only a single species or genus of bat.

In urban areas, it is expected that there will be a reduction in specialization and a consequent increase in nesting resulting from the lower availability of shelters in these areas, which would lead to an increase in the aggregation of different species of bats in a single shelter, facilitating the transmission of parasites among them (Urbieta *et al*., 2021). However, this was not observed in this study because the network obtained exhibited high specialization and low nesting. Urbieta *et al*. (2021) demonstrated that bat–ectoparasite interaction networks maintained their structural characteristics (specialization and modularity) even in areas with different levels of land use. However, Hernández-Martinez *et al*. (2018) recorded differences in the degree of specialization of networks, which were more specialized in areas with a lower degree of fragmentation.

Habitat loss can influence host–parasite interactions, affecting the survival of both groups involved, where the extinction of a given host species can cause secondary extinctions of highly specific parasites (Gómez and Nichols, 2013). In addition, vegetation loss can affect the availability of food and shelter, influencing the abundance and occurrence of hosts and altering the structure of the interaction network (Cottontail *et al*., 2009; Pilosof *et al*., 2012).

In this study, the centrality values showed low variation, with most species having values equal to 0, especially for BC. The species richness of a community determines the size of the network, which can influence characteristics such as vulnerability, generality, connectivity and link density (Tylianakis *et al*., 2007). This can indirectly affect the size of the network, favouring parasite richness (Kruess, 2003; Albrecht *et al*., 2007). In addition, species abundance can influence the frequency and detectability of interactions (Pellissier *et al*., 2013; Bartomeus *et al*., 2016) because more abundant species are more likely to interact (Vázquez *et al*., 2009; Canard *et al*., 2014). Thus, the low centrality values obtained in this study could be associated with low connectance (0.12), high specialization (0.96) and the size of the network, as it was composed of only 10 bat species and 13 ectoparasites, which could have limited the number of interactions recorded, and consequently, the centrality values.

Given the importance of ectoparasites of bats in the ecology, control and population dynamics of the host, this study provides relevant information on these topics. In addition, more studies should be conducted involving host–parasite interaction networks to improve our understanding of such networks.

## Data Availability

Not applicable.

## Appendix 1. Relationship of ectoparasites recorded in bats captured in urban green areas of Grande Aracaju, Sergipe

**Table:**

Area	Host	Ectoparasite
UFS	*Artibeus lituratus*	*Paratrichobius longicrus*
		*Trichobius* sp. (*dugesii* complex)
	*Artibeus planirostris*	*Megistopoda aranea*
		*Aspidoptera phyllostomatis*
	*Carollia perspicillata*	*Speiseria ambigua*
		*Trichobius joblingi*
	*Platyrrhinus lineatus*	*Trichobius angulatus*
	*Phyllostomus hastatus*	*Trichobius longipes*
	*Myotis lavali*	*Basilia travassosi*
	*Artibeus obscurus*	*M. aranea*
	*Sturnira lilium*	*Aspidoptera falcata*
SEFAZ	*A. lituratus*	*P. longicrus*
		*Trichobius* sp. (*dugesii* complex)
	*A. obscurus*	*A. phyllostomatis*
		*M. aranea*
	*A. planirostris*	*A. phyllostomatis*
		*M. aranea*
	*C. perspicillata*	*T. joblingi*
	*M. lavali*	*B. travassosi*
	*Phyllostomus discolor*	*Trichobioides perspicillatus*
		*Trichobius costalimai*
	*P. lineatus*	*T. angulatus*
	*S. lilium*	*A. falcata*
		*Megistopoda proxima*
VILA	*A. lituratus*	*P. longicrus*
		*Trichobius* sp. (*dugesii* complex)
	*A. obscurus*	*A. phyllostomatis*
		*M. aranea*
	*A. planirostris*	*A. phyllostomatis*
		*M. aranea*
	*P. discolor*	*T. perspicillatus*
		*T. costalimai*
	*P. lineatus*	*T. angulatus*
	*S. lilium*	*A. falcata*
		*Megistopoda proxima*
	*Myotis riparius*	*B. travassosi*
	*M. lavali*	*B. travassosi*
